# Supplementary material for: A Maize Male Gametophyte-Specific Gene Encodes ZmLARP6c1, a Potential RNA-Binding Protein Required for Competitive Pollen Tube Growth
Source: Front Plant Sci. 2021 Feb 25;12:635244. doi: 10.3389/fpls.2021.635244 (PMC7947365; doi:10.3389/fpls.2021.635244)

Supplementary Figure 1

A

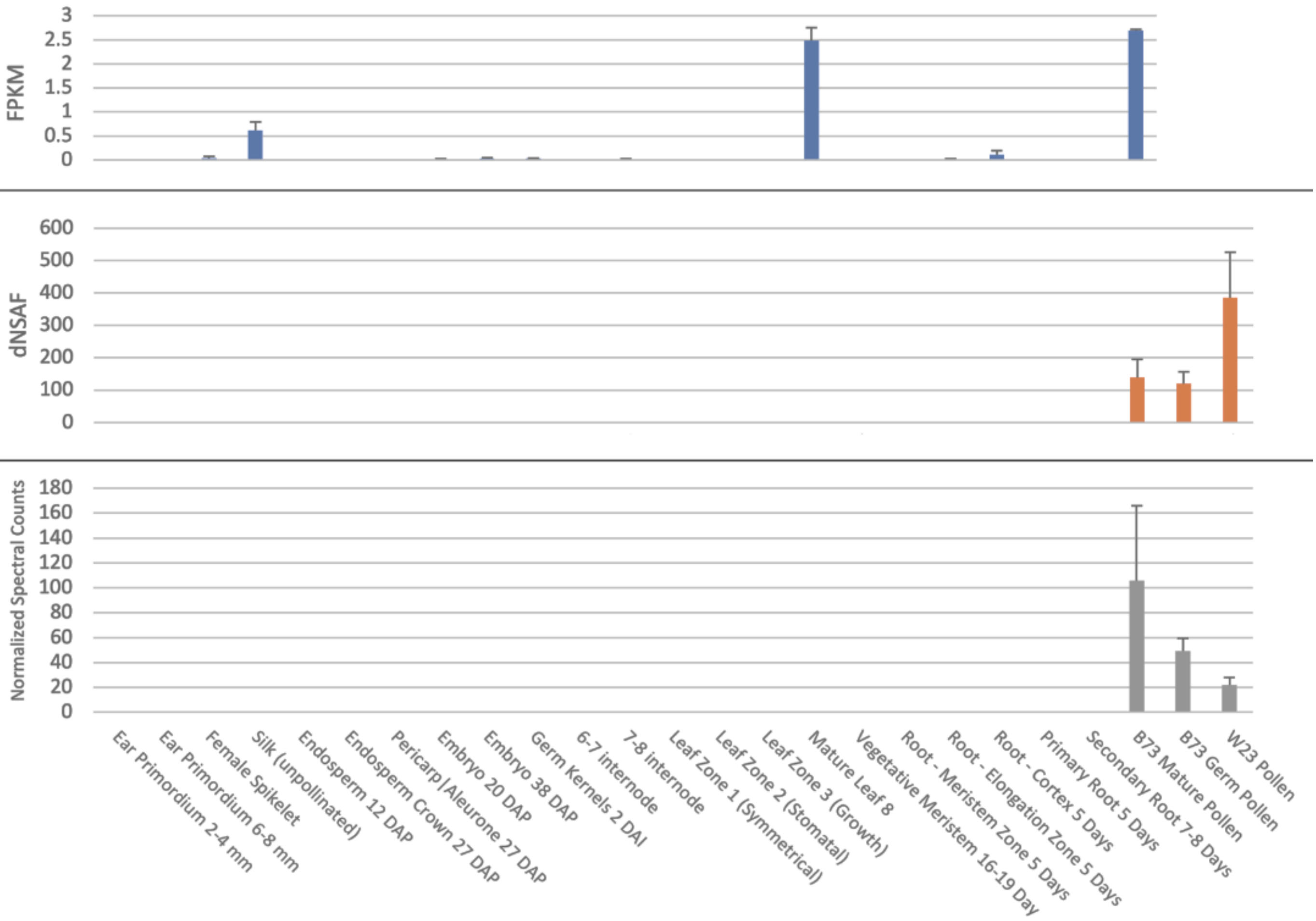

B

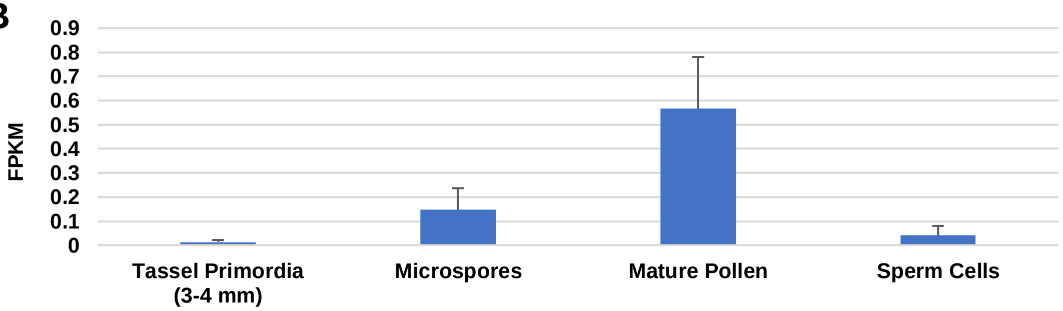

Supplementary Figure 2

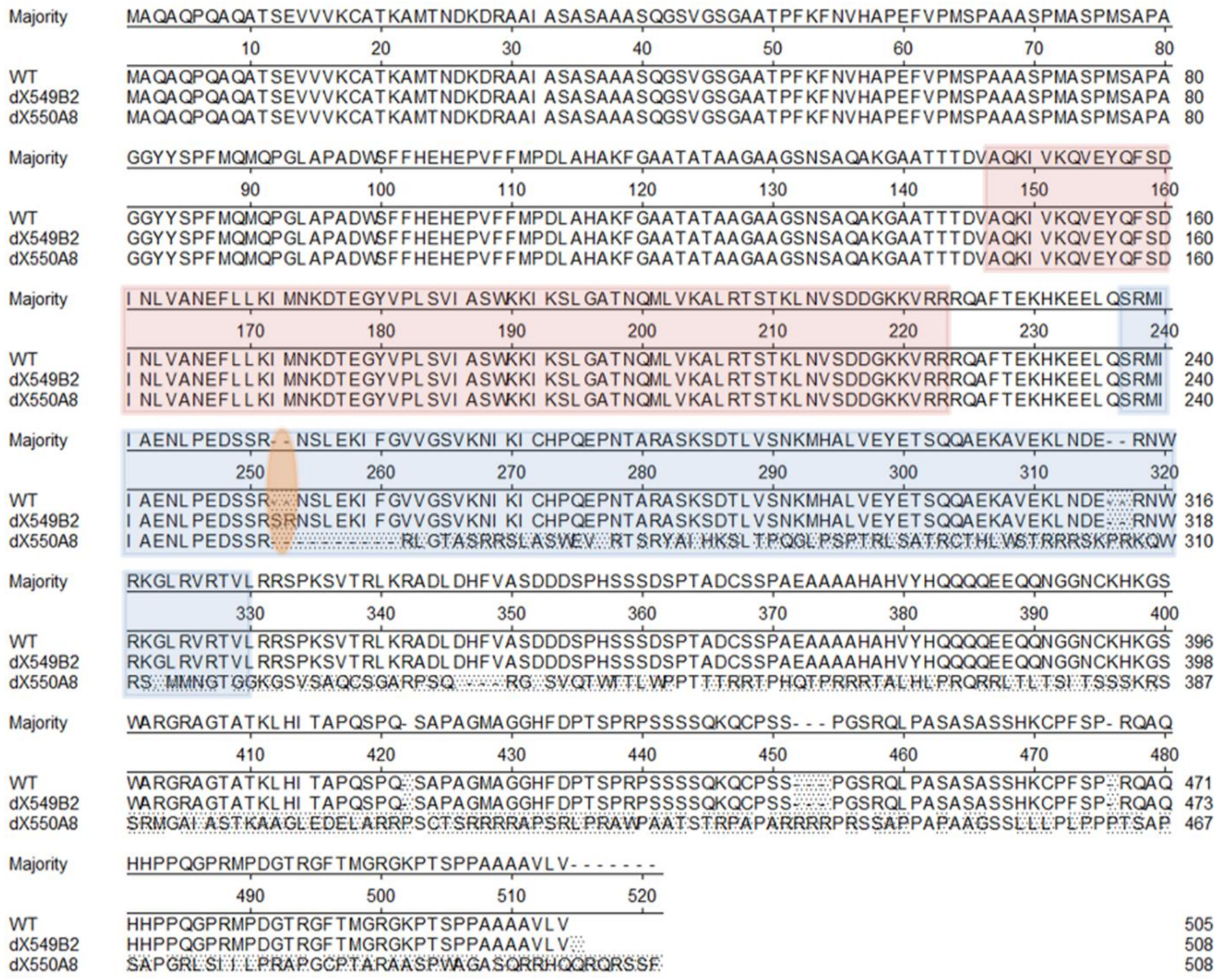

Supplementary Figure 3

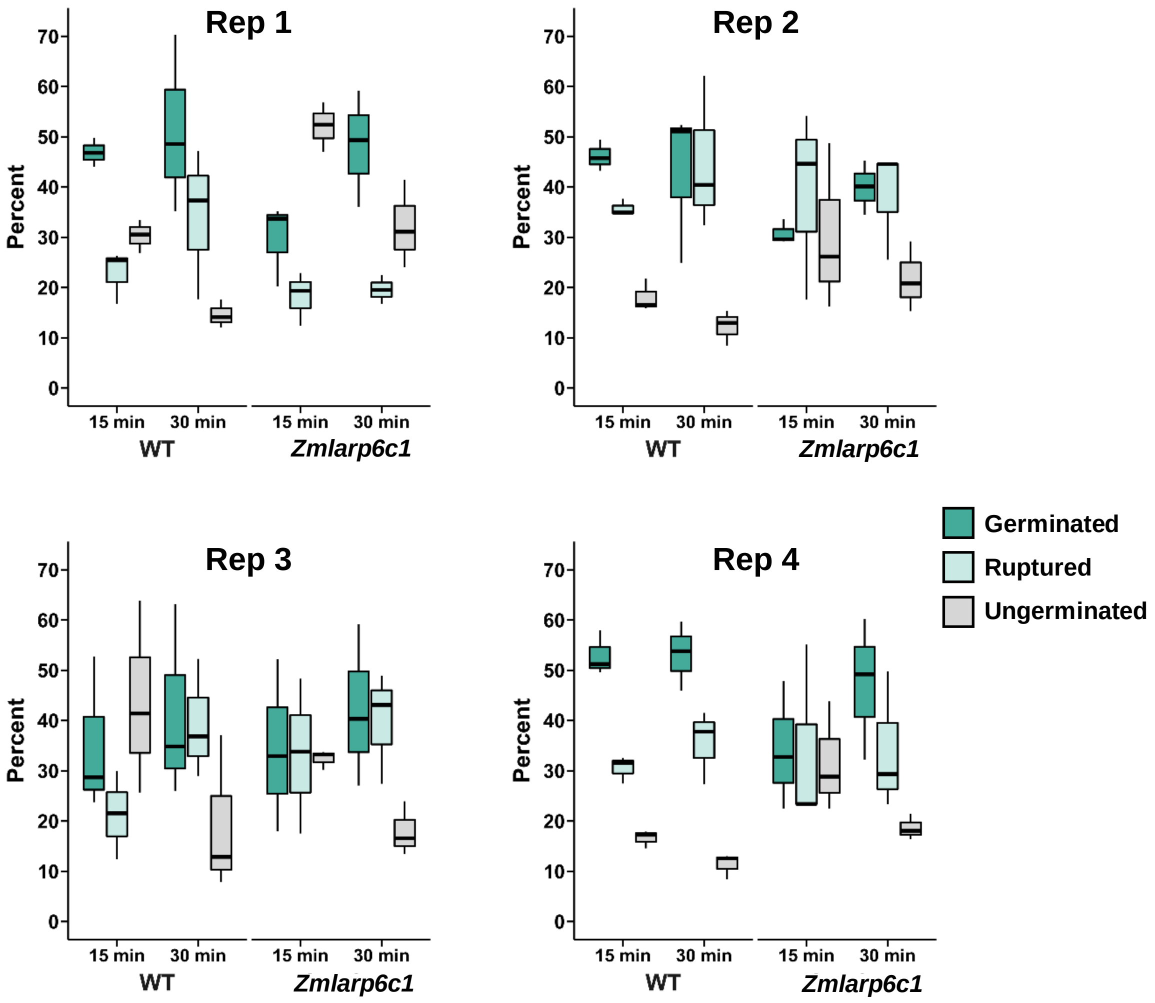

Supplementary Figure 4

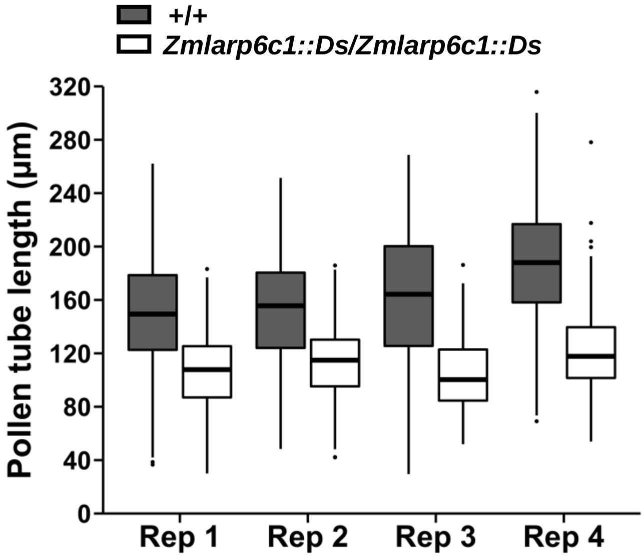

Supplementary Figure 5

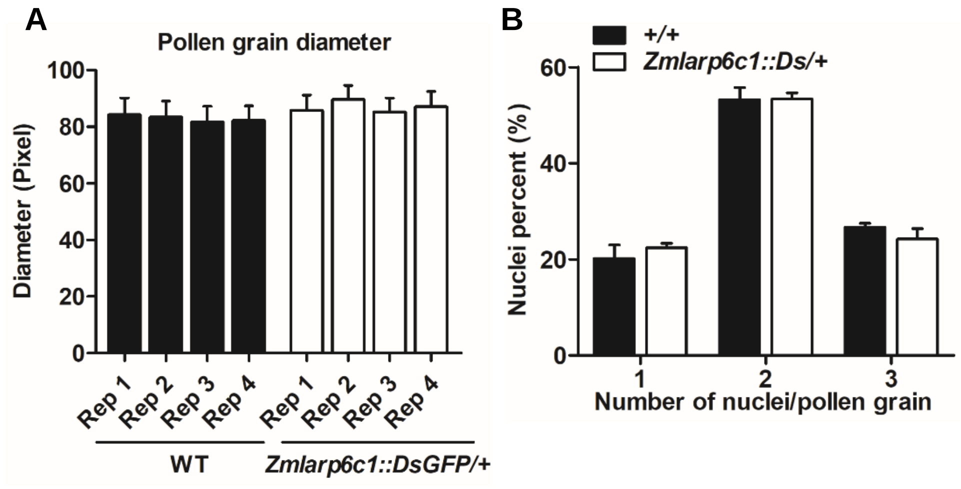

Supplement: Supplementary Figure 1 — Zmlarp6c1 expression is enriched specifically in mature pollen. (A) Expression profiling data from the Walley et al. (2016) maize developmental atlas, for GRMZM2G323499/Zm00001d018613. RNA-seq data for all 23 different tissue types assessed (blue), with corresponding proteomic (orange), and phosphoproteomic (gray) profiling data for the same tissues. Two additional, relevant samples were assessed for proteomic data: B73 (inbred line) Germinated Pollen and W23 (inbred line) Mature Pollen. Although Zmlarp6c1 transcript is detected in mature leaf, the translated protein is only detected in pollen samples. FPKM, fragments per kilobase of exon model per million reads mapped; dNSAF, distributed normalized spectral abundance factor. (B) Focused transcriptome profiling of maize male gametophyte development (Warman et al., 2020) indicates that the Zmlarp6c1 transcript (Zm00001d018613_T004) is highest in mature pollen, but largely excluded from the sperm cells isolated from mature pollen. This suggests the transcript is specifically enriched in the pollen vegetative cell, which drives pollen tube germination and growth. [file Image_1.pdf]
